# Joint Hypermobility as a Potential Indicator of Marfan Syndrome and Ehlers-Danlos Syndrome

**DOI:** 10.7759/cureus.27574

**Published:** 2022-08-01

**Authors:** Henry Zou, Philip Waalkes

**Affiliations:** 1 Family Medicine, Michigan State University College of Human Medicine, Grand Rapids, USA; 2 Family Medicine, Spectrum Health Medical Group, Zeeland, USA

**Keywords:** genetic screening, genetic analysis, joint hyperlaxity, ehlers danlos syndrome, marfan disease

## Abstract

Marfan syndrome (MFS) is an autosomal dominant connective tissue disorder associated with the mutation of the *FBN1* gene. Ehlers-Danlos syndrome (EDS) is a group of inherited connective tissue disorders with similar clinical features to MFS that often requires genetic testing to confirm a diagnosis of an EDS subtype. MFS is diagnosed using the Ghent Nosology criteria, which screens for cardiovascular, musculoskeletal, integumentary, ocular, and pulmonary abnormalities. Though genetic testing has recently been increasingly emphasized in diagnosing MFS, it is not currently a mandatory component of the Ghent Nosology. We present the case of a nine-year-old male who presented with joint hypermobility of the shoulders, knees, and thumbs, and a family history of joint hypermobility in his 15-year-old brother. Genetic testing ruled out MFS, and the patient subsequently underwent testing for EDS, which further ruled out classical and hypermobile EDS. This case highlights the importance of supplementing the Ghent Nosology criteria with genetic testing in diagnosing MFS because it can aid in generating a differential diagnosis and optimizing diagnostic accuracy.

## Introduction

Marfan syndrome (MFS) is an autosomal dominant genetic disorder that affects connective tissue, specifically the synthesis of elastin [1]. This rare disorder affects 1/5,000 individuals and is known to present with variable expressivity [1]. MFS is associated with a mutation of the fibrillin 1 (*FBN1*) gene on chromosome 15 [1]. As a secondary effect of this mutation, increased levels of transforming growth factor β (TGF-β) can manifest as abnormalities in aortic and pulmonary vasculature [2]. MFS is diagnosed using standardized clinical criteria known as the Ghent Nosology, including musculoskeletal, cardiovascular, integumentary, ocular, and pulmonary abnormalities [3,4]. The Ghent Nosology differentiates between “major” and “minor” manifestations of organ system abnormalities, and a formal diagnosis of an index patient requires the major involvement of at least two organ systems and the minor involvement of a third organ system [3]. Though the Ghent Nosology combined with molecular techniques can confirm the presence of FBN1 mutations in 97% of patients who fulfill the MFS diagnosis criteria, concerns remain on whether the criteria have been sufficiently validated and are fully applicable to pediatric patients [3]. Updated scoring systems have placed greater diagnostic importance on FBN1 testing; nonetheless, genetic testing is still not a mandatory component of Ghent Nosology criteria [3].

Ehlers-Danlos syndrome (EDS) refers to a heterogeneous group of inherited connective tissue disorders that encompasses 13 subtypes; some subtypes are autosomal dominant, while others are autosomal recessive [5]. EDS can display many similar clinical features to MFS, including joint hypermobility, abnormal bruising or bleeding, vessel rupture or dissection, and hollow organ rupture [6]. Diagnostic criteria vary widely between EDS subtypes; though electron microscopy of skin biopsies and urine testing may be useful first steps to screen for certain subtypes, diagnostic confirmation typically requires genetic testing [6].

We present the case of a pediatric patient who displayed symptoms initially suggestive of MFS, which illustrates the importance of supplementing Ghent Nosology criteria with genetic testing in diagnosis.

This article was previously presented as a poster at the Inaugural Orthopedic Surgery Research Symposium for Medical Students in Michigan on December 1, 2021. It was also presented as a poster at the Early Clinical Experience Scholarly Project Symposium at the Michigan State University College of Human Medicine on March 1, 2022.

## Case presentation

**Figure 1:**
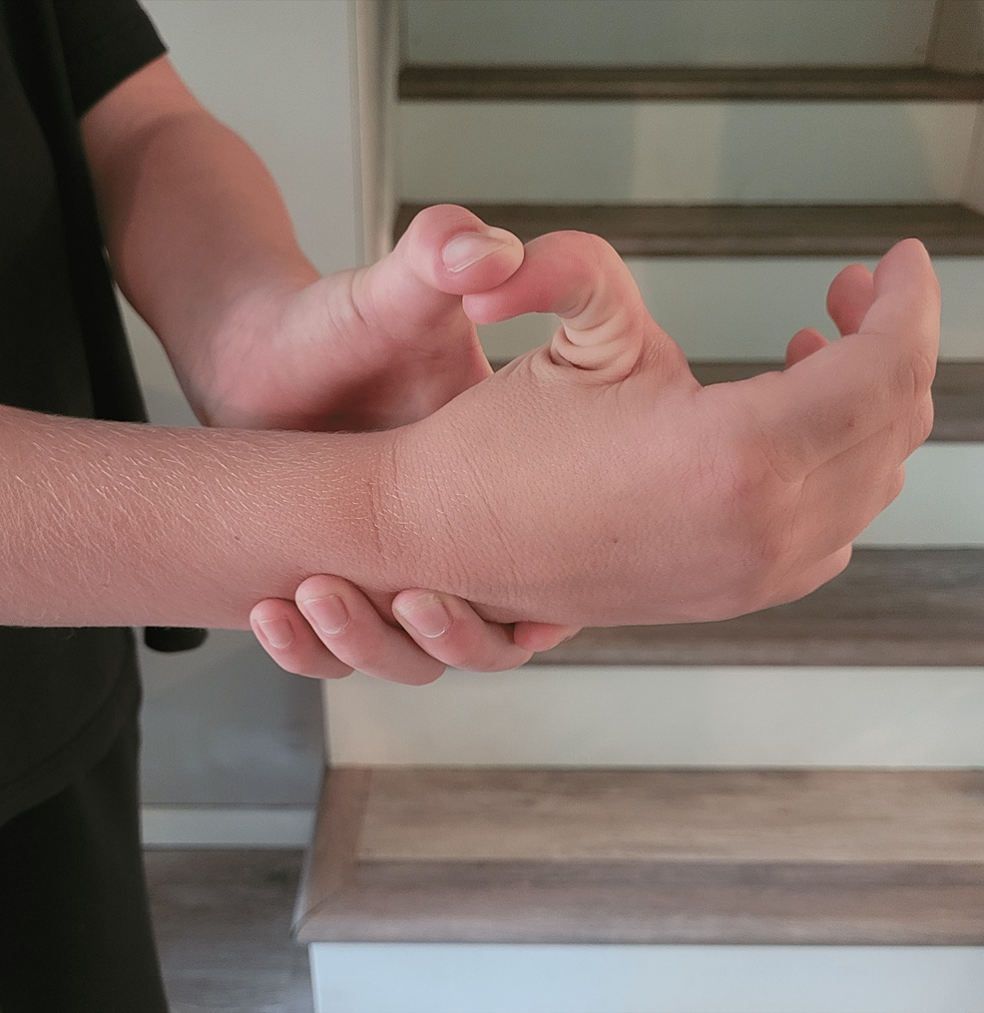
Hyperextensibility of the thumb at the interphalangeal joint Published with permission from the patient and his legal guardian.

**Figure 2:**
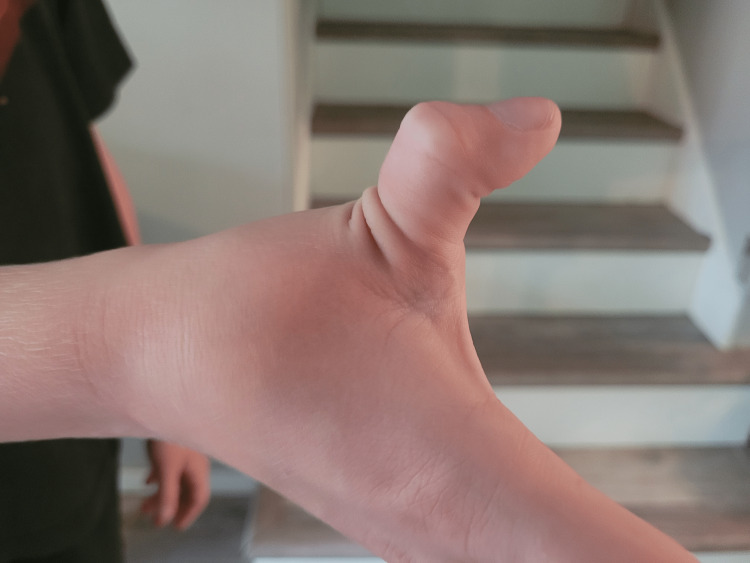
Hyperextensibility of the thumb at the metacarpophalangeal joint Published with permission from the patient and his legal guardian.

A nine-year-old male with a history of mild intermittent asthma without complications presented with his father for a routine well-child examination. The father reported concerns about the patient being “very flexible,” as he could pop his shoulders out of their joints bilaterally, crack his neck, demonstrate hyperflexible knees bilaterally, and bend his thumb to his wrist without pain (Figures 1, 2). The patient denied chest pain or vision symptoms; the father reported that the patient’s 15-year-old older brother demonstrated similar joint hypermobility but to a lesser extent. The patient met all developmental milestone criteria for his age range of 9-10 years. His vital signs were within normal limits; his height was in the 89th percentile, weight was in the 78th percentile, and BMI was in the 60th percentile according to the CDC criteria for boys aged 2-20 years. His physical examination was unremarkable except for the musculoskeletal exam; he could dislocate and relocate both shoulders, extend both thumbs almost 180 degrees, and had lax anterior cruciate ligaments (ACL) and posterior cruciate ligaments (PCL) bilaterally.

The patient was diagnosed with joint disorders of multiple sites and referred for evaluation of MFS; however, genetic testing ruled out MFS. The patient was scheduled to undergo testing for EDS, and subsequent tests ruled out classical and hypermobile EDS. The patient and his family were appropriately counseled.

## Discussion

This case illustrates the relevance of assigning priority to genetic testing in the process of diagnosing MFS. Though the patient displayed joint hypermobility, a symptom classically associated with MFS, he did not meet the Ghent Nosology criteria required for formal MFS diagnosis as he did not display symptoms concurrent with the major involvement of two or more organ systems. Furthermore, joint hypermobility was removed from the updated 2010 Ghent Nosology criteria due to a perceived lack of specificity to MFS pathophysiology [7]. Regarding the validity of solely relying on Ghent Nosology criteria and potential changes to the criteria, this case highlights how genetic testing can not only help confirm/rule out MFS but also aid in generating a differential diagnosis such as EDS.

This case emphasizes the importance of genetic testing for MFS and EDS as diagnostic criteria (Table 1) [1,5,8]. There are concerns surrounding the diagnostic validity of Ghent Nosology criteria for pediatric patients, significant variations to the criteria over time, and improvements in molecular techniques [3]. Furthermore, as a diagnosis of MFS or EDS can be a significant psychosocial stressor, hinder access to life insurance and career/lifestyle opportunities, and necessitate longitudinal clinical monitoring, incorporating genetic testing to optimize diagnostic accuracy and reduce the risk of false positives can significantly impact a patient’s quality of life [3,5,6].

**Table 1:** Genetic screening for Marfan syndrome and Ehlers-Danlos syndrome

Disorder	Subtype	Gene(s) screened during testing
Marfan syndrome	N/A	FBN1
Ehlers-Danlos syndrome	Classical	COL5A1, COL5A2, COL1A1
	Classical-like	TNXB
	Cardiac-valvular	COL1A2
	Vascular	COL3A1, COL1A1
	Hypermobile	Unknown
	Arthrochalasia	COL1A1, COL1A2
	Dermatosparaxis	ADAMTS2
	Kyphoscoliotic	PLOD1, FKBP14
	Brittle cornea syndrome	ZNF469, PRDM5
	Spondylodysplastic	B4GALT7, B3GALT6, SLC39A13
	Musculocontractural	CHST14, DSE
	Myopathic	COL12A1
	Periodontal	C1R

## Conclusions

Genetic testing is often vital in diagnosing EDS subtypes and providing a definitive diagnosis for MFS. However, it is not currently a mandatory component of the Ghent Nosology criteria used to diagnose MFS. This case highlights the importance of including genetic testing in optimizing diagnostic accuracy for patients who present with clinical features consistent with MFS and EDS.
